# Involvement of Iron-Evoked Oxidative Stress in Smoking-Related Endothelial Dysfunction in Healthy Young Men

**DOI:** 10.1371/journal.pone.0089433

**Published:** 2014-02-21

**Authors:** Kei Fukami, Sho-ichi Yamagishi, Shuji Iida, Hidehiro Matsuoka, Seiya Okuda

**Affiliations:** 1 Division of Nephrology, Department of Medicine, Kurume University School of Medicine, Kurume City, Fukuoka, Japan; 2 Department of Pathophysiology and Therapeutics of Diabetic Vascular Complications, Kurume University School of Medicine, Kurume City, Fukuoka, Japan; 3 Division of Cardio-Vascular Medicine, Department of Medicine, Kurume University School of Medicine, Kurume City, Fukuoka, Japan; Temple University, United States of America

## Abstract

**Background:**

Oxidative stress and smoking contribute to endothelial dysfunction. Iron might also play a role in oxidative stress generation and endothelial dysfunction. However, the involvement of iron in smoking-induced endothelial dysfunction in healthy smokers remains unclear. Therefore, we examined here whether (1) intravenous iron infusion impaired endothelial function evaluated by flow-mediated vasodilatation (FMD) in non-smokers, and (2) deferoxamine, a potent iron chelator, ameliorated endothelial dysfunction in healthy smokers.

**Methods:**

Eight healthy young male non-smokers (23±4 years old) received intravenous injection of saccharated ferric oxide (0.7 mg/kg body weight), while 10 age-matched healthy male smokers received deferoxamine mesylate (8.3 mg/kg body weight). At baseline, 5 and 20 minutes after treatment with iron or deferoxamine, biochemical variables were measured, including serum iron and marondialdehyde (MDA), a marker of lipid oxidation, and endothelial function was simultaneously evaluated by FMD.

**Results:**

Compared with non-smokers, FMD was significantly lower in smokers. Iron and MDA levels were significantly increased, whereas FMD was impaired by iron infusion in non-smokers. Conversely, deferoxamine treatment significantly decreased iron and MDA levels and restored the decreased FMD in smokers. Baseline serum iron and MDA levels in all 18 subjects (non-smokers and smokers) were correlated with each other. There was a significant inverse correlation between the changes in MDA values and FMD from baseline in 18 men. Endothelium-independent vasodilation by glyceryl trinitrate was unaltered by either treatment.

**Conclusions:**

Our present study suggests that iron-evoked oxidative stress might play a role in endothelial dysfunction in healthy smokers.

## Introduction

In normal vascular physiology, endothelium-derived nitric oxide (NO) is the most potent endogenous vasodilator and, as a result of its anti-inflammatory, anti-proliferation, and anti-thrombotic effects, it is widely recognised as an endogenous anti-atherogenic factor [1], [2]. Indeed, endothelial dysfunction due to the reduced NO production and/or bioavailability is a common feature in patients with apparent coronary atherosclerosis, smokers or in high risk-patients with dyslipidaemia or diabetes [1]–[5]. It is also considered to be an initial step in the course of atherosclerotic cardiovascular disease (CVD) [1]–[5]. Although there is accumulating evidence that oxidative stress plays a pivotal role in endothelial dysfunction [3], [6]–[8] and that smokers show increased markers of oxidative stress [9], [10], the underlying molecular mechanism for smoking-induced endothelial dysfunction in healthy smokers is not fully understood.

Iron is an essential metal that can contribute not only to the storage and transport of oxygen, but is also required to catalyse various redox reactions [11]. Most of the iron in the body is found in haemoglobin within erythrocytes and stored in macrophages and the liver as ferritin and haemosiderin [12]. Although cellular iron haemostasis is finely regulated in the body [13], iron excess can occur; it is highly toxic and causes tissue damage by stimulating free radical oxidations such as lipid peroxidation via the Fenton reaction [14]. Indeed, several epidemiological studies have suggested the link between iron overload and CVD [3], [14], [15]. Atomic absorption spectrometry has revealed that serum iron is significantly higher in patients with coronary heart disease compared with healthy controls [15]. Serum iron levels are positively associated with the risk of fatal acute myocardial infarction [3]. Furthermore, high levels of stored iron, assessed by serum ferritin levels, have been shown to be a strong and independent risk factor for acute myocardial infarction in Finnish men without symptomatic coronary heart disease [4]. However, it remains unclear whether iron is directly involved in endothelial dysfunction in both healthy non-smokers and smokers.

Therefore, in this study, we examined whether (1) intravenous iron infusion could actually impair endothelial function evaluated by flow-mediated vasodilatation (FMD) in young healthy male non-smokers and (2) deferoxamine, a potent iron chelator, is able to ameliorate endothelial dysfunction in age- and sex-matched healthy smokers.

## Materials and Methods

### Subjects and General Procedure

#### Iron infusion study

Eight healthy young male non-smokers (mean age, 23±4 years old; 19 to 31 years old), who were non-obese (body mass index, 22.4±1.7 kg/m^2^), normotensive (systolic/diastolic blood pressure, 118±7/72±8 mmHg) and normoglycaemia (fasting plasma glucose, 88±11 mg/dl) were enrolled in the iron infusion study. Blood was drawn after 12-hour fasting for the determination of clinical variables. Then, saccharated ferric oxide (iron; 0.7 mg/kg body weight) (Nichiiko Co., Toyama, Japan) or iron-free 5% glucose solution, as a control, was intravenously injected for 20 min. Five and twenty min after the procedure, blood samples were obtained again for the measurements of serum iron and malondialdehyde (MDA) levels. FMD was measured before and after the administration of iron. Just after the measurement of FMD, endothelium-independent vasodilatation was evaluated with the sublingual administration of glyceryl trinitrate (GTN). Control experiments were performed first. At least 7 days after, the iron infusion study was performed in the same 8 non-smokers.

#### Iron chelation study

In the iron chelation study, 10 age-matched healthy male smokers (mean age, 23±1 years old; range, 21 to 25 years old) were enrolled. Baseline blood chemistries were measured under the fasting conditions. Then, deferoxamine mesylate (8.3 mg/kg body weight) (Novartis Pharma Co., Tokyo, Japan) or isotonic sodium chloride solution, as a control, were administrated for 20 min. As in the iron infusion study, blood samples were obtained 5 and 20 min after the exposure and serum iron and MDA levels were measured. FMD and GTN-induced vasodilatation were evaluated as described above.

Written informed consent was obtained from all subjects. The study was approved by an ethics committee of the Kurume University School of Medicine, Japan (UMIN 000012156).

### Data Collection

Medical history was ascertained by a questionnaire. Blood pressure was measured in the supine position using an upright standard sphygmomanometer. Fasting blood was drawn from a superficial vein of the left arm just before the infusion of iron, deferoxamine or control solutions for the determinations of haemoglobin, total cholesterol, triglycerides, high-density lipoprotein cholesterol, plasma glucose, glycated haemoglobin (HbA1c), serum iron, total iron binding capacity and ferritin. Low-density lipoprotein cholesterol was calculated using the Friedewald formula [16]. Transferrin saturation was determined as the ratio of serum iron and total iron-binding capacity, multiplied by 100. Serum cotinine levels were determined by enzyme-linked immunosorbent assay kits (Kainos Laboratories, Inc., Tokyo, Japan). MDA levels were measured by thiobarbituric acid-reactive substance assay kits (Wako Chemicals Co., Osaka, Japan). Blood chemistries were measured at a commercially available laboratory (SRL Inc., Hachioji, Japan).

### Measurements of Endothelium-dependent and Independent Vasodilatation

Endothelial function was assessed by the non-invasive measurement of flow-mediated vasodilatation, as described previously [17]. In brief, after a 10-min equilibration period in the supine position, the brachial artery was longitudinally imaged approximately 5 cm proximal to the antecubital crease with the use of a 10-MHz linear-array transducer and the SSA-380A system (Toshiba Co., Osaka, Japan). After recording the baseline arterial diameter, the upper arm was occluded at 250 mmHg of pressure with a 12.5-cm-wide cuff for 4.5 min, and then released. The images of the brachial artery were continuously recorded for 15 min after releasing the pressure. Photographic images of end-diastolic frames were obtained and diameter was measured by a calliper at the single most equivalently imaged site with side-by-side presentation, with 2 independent investigators blinded to the subjects and sequences. FMD, i.e. endothelium-dependent vasodilatation, was determined as the maximal percent diameter change of the post-occlusion arterial diameter measurement relative to the mean of the corresponding 2 baseline measurements. The inter-observer and intra-observer variations were 2.8% and 1.6%, respectively. As an internal control, endothelium-independent vasodilatation was induced by sublingual glyceryl trinitrate (GTN; 300 mg; Myocol Spray, Toa Eiyo Co., Tokyo, Japan) after the measurement of FMD and changes in diameter from the baseline were assessed.

### Statistical Analysis

A one-way analysis of variance (ANOVA) for repeated measures was used to assess the changes in serum iron and MDA levels. To compare clinical valuables between healthy non-smokers and smokers, an unpaired *t*-test was performed. To examine the differences in FMD and GTN-induced vasodilatation before and after the administration of iron, deferoxamine or control, the paired *t*-test was used. To determine the correlation between serum iron and MDA levels at baseline and changes in MDA values and FMD from baseline (delta (Δ)MDA and ΔFMD), linear regression analysis was performed. All values are expressed as the mean values ± standard deviation, and p<0.05 was considered to be statistically significant. All statistical analyses were performed using the SPSS system (Ver. 20, SPSS, Chicago, IL, USA).

## Results

### Demographic Data in Smokers and Healthy Non-smokers

Demographic baseline data are shown in Table 1. All subjects were non-obese, normotensive and normoglycaemic. Triglyceride levels tended to be higher in smokers than in healthy non-smokers (104±53 mg/dl vs. 59±23 mg/dl, p = 0.062). Although there was no significant difference in serum iron and MDA levels between the two groups, FMD values in smokers were significantly decreased compared with non-smokers (4.6±1.4% vs. 9.8±4.1%, p = 0.008, Table 1). GTN-induced vasodilatation did not differ between the two groups (24.2±5.0% vs. 22.0±2.1%, p = 0.398, Table 1).

**Table 1:** Clinical characteristics of the subjects.

	Healthy non-smokers	Smokers	95% CI	p-value
Number of patients	8	10		
Age (years)	23±4	23±1	−2.90, 3.55	0.822
Body Mass Index (kg/m^2^)	22.4±1.7	23.1±3.0	−3.09, 1.68	0.536
Systolic BP (mmHg)	118±7	114±9	−4.55, 11.55	0.371
Diastolic BP (mmHg)	72±8	69±6	−4.56, 9.41	0.472
Haemoglobin (g/dl)	14.8±0.3	15.1±1.1	−1.12, 0.55	0.472
Total cholesterol (mg/dl)	148±18	161±34	−44.15, 18.11	0.383
LDL-cholesterol (mg/dl)	82±9	94±24	−33.41, 9.41	0.246
Triglycerides (mg/dl)	59±23	104±53	−90.69, 2.55	0.062
HDL-cholesterol (mg/dl)	54±11	50±9.6	−7.38, 15.09	0.472
FPG (mg/dl)	88±11	98±9	−19.85, 1.01	0.074
HbA1c (%)	5.0±0.2	4.9±0.2	−0.09, 0.28	0.273
Serum cotinine (ng/ml)	not detected	120±72		
Serum iron (mg/dl)	93±39	105±51	−59.12, 33.92	0.574
TSAT (%)	30.4±12.1	35.0±16.7	−19.79, 10.17	0.506
Ferritin (ng/ml)	112±70	121±66	−79.02, 60.74	0.784
Serum MDA (nmol/l)	2.46±0.50	2.49±0.74	−0.68, 0.62	0.929
**FMD (%)**	**9.8±4.1**	**4.6±1.4**	**1.79, 8.68**	**0.008**
GTN-induced VD (%)	22.0±2.1	24.2±5.0	−7.49, 3.13	0.398

Values are shown as mean±SD. CI = confidence interval; BP = blood pressure; LDL = low-density lipoprotein; HDL = high-density lipoprotein; FPG = fasting plasma glucose; HbA1c = glycated haemoglobin; TSAT = transferrin saturation; MDA = malondialdehyde; FMD = flow-mediated vasodilatation; GTN = glyceryl trinitrate; VD = vasodilatation.

**Figure 1:**
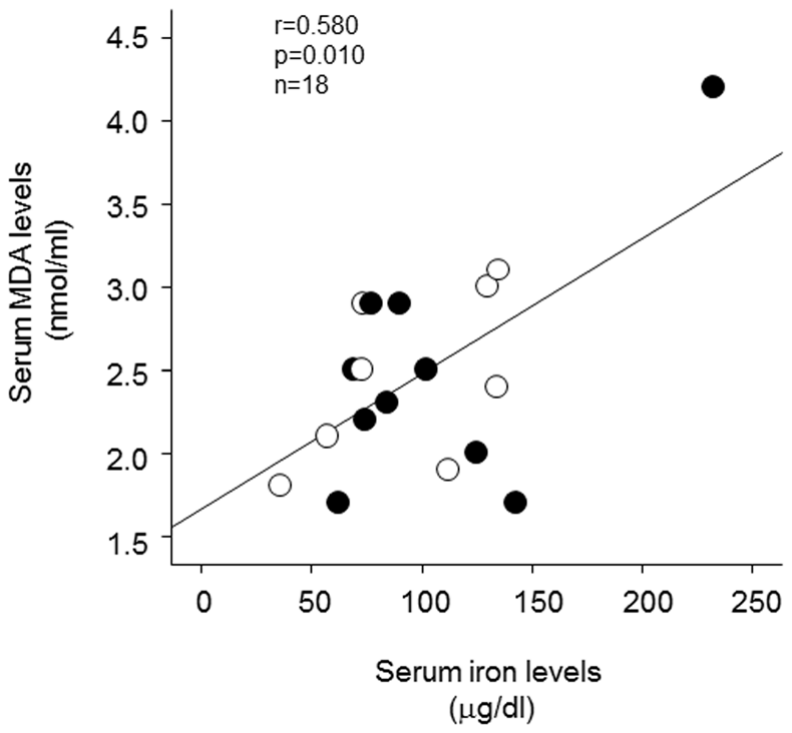
Correlation between serum iron and MDA levels at baseline in both smokers and non-smokers. Black circle; smokers, white circle; non-smokers. n = 18, r = 0.580, p = 0.010.

Linear regression analysis revealed that serum levels of iron were positively correlated with serum MDA values at baseline in both non-smokers and smokers (r = 0.580, p = 0.010, Figure 1).

### Effects of Intravenous Saccharated Ferric Oxide Infusion on Endothelial Function in Healthy Non-smokers

**Figure 2:**
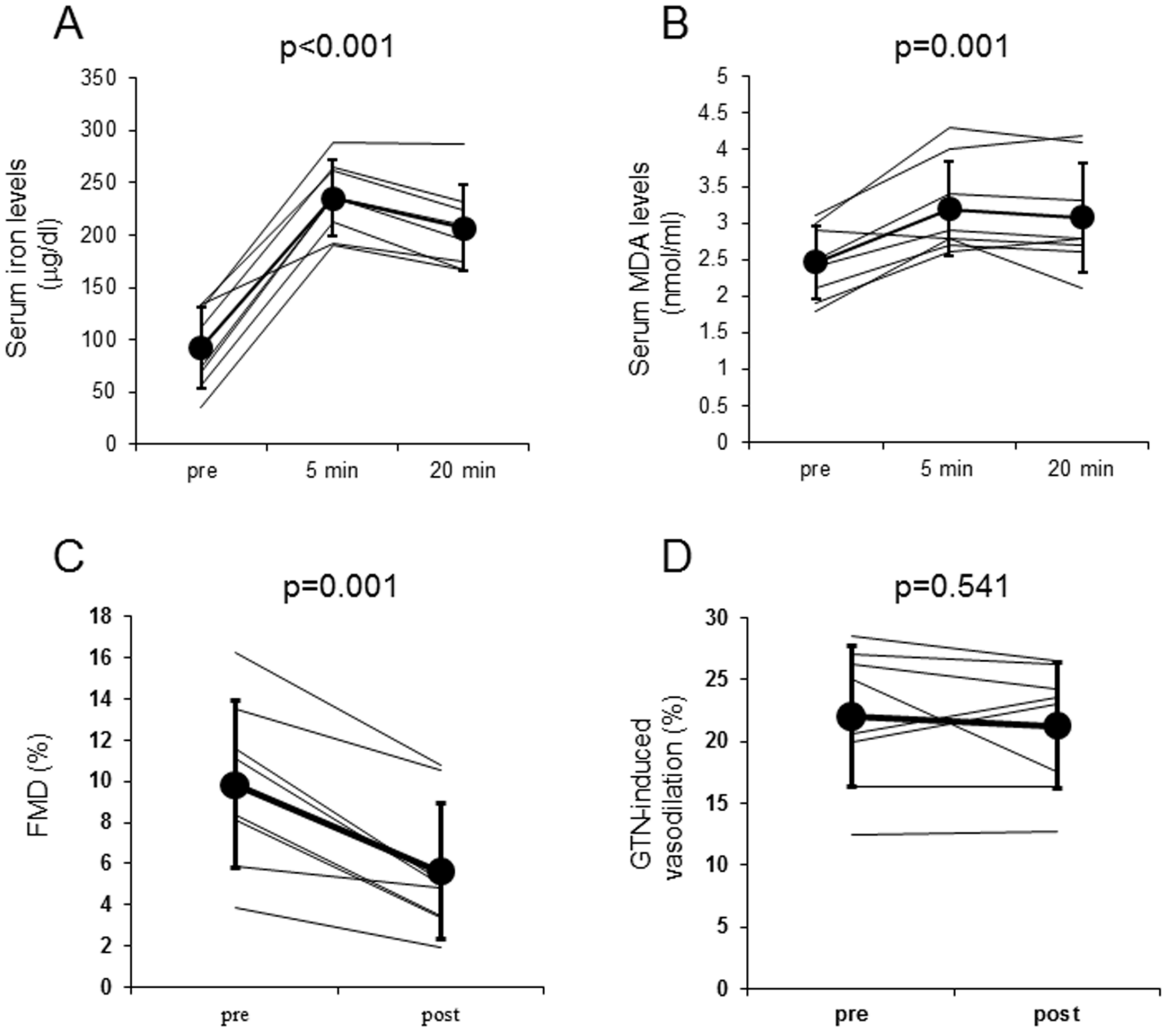
Effects of iron infusion on serum iron (A), MDA (B), FMD (C) and GTN-induced vasodilatation (D). Serum iron (A) and MDA levels (B) at baseline, 5 and 20 min after the intravenous saccharated ferric oxide infusion in non-smokers. FMD (C) and GTN-induced vasodilatation (D) before and after the intravenous saccharated ferric oxide injection.

Serum iron and MDA levels were significantly increased after the intravenous infusion of saccharated ferric oxide in healthy non-smokers (iron: before −93±39 mg/dl, after 5 min −236±36 mg/dl, after 20 min −207±41 mg/dl, p<0.001, Figure 2A; MDA: before −2.46±0.50 nmol/ml, after 5 min −3.19±0.65 nmol/ml, after 20 min −3.08±0.74 nmol/ml, p = 0.001, Figure 2B). On the contrary, FMD values were significantly decreased by the administration of iron (before −9.8±4.1%, after −5.6±3.3%, p = 0.001, Figure 2C). Iron infusion did not affect the GTN-induced vasodilatation (Figure 2D). Control treatments also did not affect serum iron, MDA levels, FMD or GTN-induced vasodilatation (data not shown).

### Effects of Iron Chelation on Endothelial Function in Smokers

**Figure 3:**
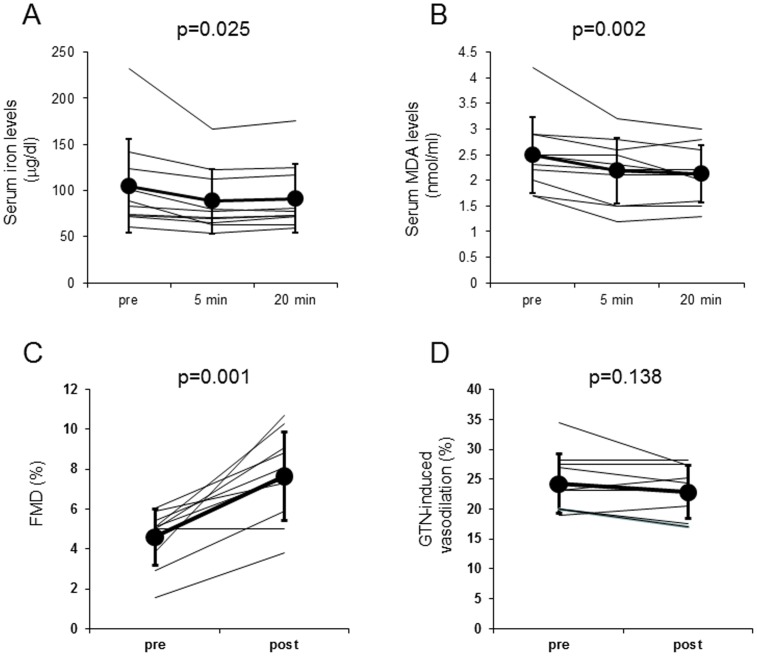
Effects of deferoxamine mesylate on serum iron (A), MDA (B), FMD (C) and GTN-induced vasodilatation (D). Serum iron (A) and MDA levels (B) at baseline, 5 and 20 min after the intravenous deferoxamine mesylate infusion in smokers. FMD (C) and GTN-induced vasodilatation (D) before and after the intravenous deferoxamine mesylate injection.

As shown in Figure 3A and B, the administration of deferoxamine mesylate significantly decreased serum iron and MDA levels in smokers (iron: before −105.1±51.2 mg/dl, after 5 min −88.3±35.3 mg/dl, after 20 min −91.6±36.8 mg/dl, p = 0.025, MDA: before −2.49±0.74 nmol/ml, after 5 min −2.19±0.63 nmol/ml, after 20 min −2.12±0.56 nmol/ml, p = 0.002, respectively). Furthermore, iron chelation by deferoxamine mesylate significantly improved FMD (before −4.6±1.4%, after −7.7±2.2%, p = 0.001, Figure 3C). Deferoxamine mesylate treatment did not affect the GTN-induced vasodilatation (Figure 3D). Serum iron, MDA levels, FMD or GTN-induced vasodilatation was not changed by the infusion of control sodium chloride solution (data not shown).

**Figure 4:**
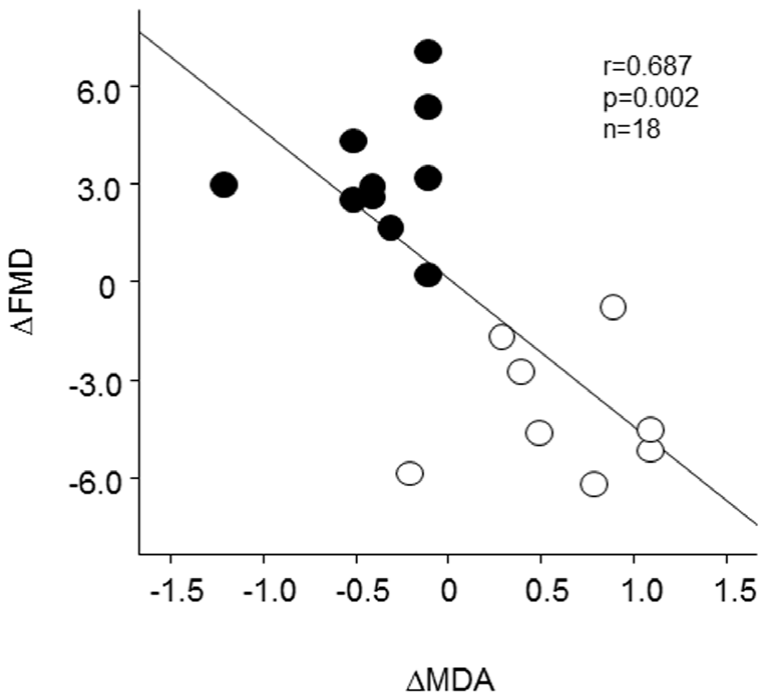
Correlation between ΔFMD and ΔMDA in total 18 subjects. Black circle; smokers, white circle; non-smokers. n = 18, r = 0.687, p = 0.002.

In addition, there was a significant inverse correlation between ΔMDA and ΔFMD in all 18 subjects (r = 0.687, p = 0.002, Figure 4).

## Discussion

The salient findings of this study are as follows: (1) there was a positive correlation between serum iron and MDA levels at baseline in both healthy smokers and non-smokers, (2) endothelial function evaluated by FMD was significantly impaired in healthy young smokers compared with age- and sex-matched healthy non-smokers, (3) iron infusion significantly impaired endothelial function in non-smokers, which was associated with the increase in serum iron and MDA levels, (4) iron chelation by deferoxamine significantly decreased serum iron and MDA values and improved endothelial dysfunction in smokers, (5) there was a significant inverse association between the changes in serum MDA levels and FMD in all 18 subjects, and (6) iron or deferoxamine treatment did not affect endothelium-independent vasodilation by GTN. These observations suggest that, although in this study there was no significant difference of serum iron levels between healthy young non-smokers and smokers, iron-evoked oxidative stress might contribute to smoking-related endothelial dysfunction in healthy male smokers.

The present findings observed in healthy non-smoker controls have extended the previous observation which showed that intravenous ferric saccharate supplementation induced superoxide generation in white blood cells and impaired FMD in 20 healthy volunteers [18]. However, there was some controversy regarding the effects of iron on MDA values and FMD. Indeed, a couple of papers reported that although intravenous infusion of iron significantly increased fasting plasma MDA levels [19], it *did not* impair endothelial function in dialysis patients [20]–[22]. Therefore, the role of iron and iron-evoked oxidative stress generation in endothelial dysfunction might differ depending on the patients’ clinical background. However, several epidemiological studies have shown that iron overload is associated with CVD [14], [15]. Furthermore, two independent groups showed that intima-media thickness of the carotid artery, a marker of atherosclerosis, was associated with oxidative stress markers, serum ferritin or intravenous iron dose administered in haemodialysis patients [23], [24]. These observations indicate that intravenous iron therapy or iron overload could increase the incidence of CVD, partly via oxidative stress generation.

In this study, we found for the first time that deferoxamine treatment significantly decreased serum levels of iron and MDA and restored the impairment of endothelial function in healthy, young male smokers. Iron chelation by deferoxamine has been shown to improve endothelial function in patients with coronary artery disease [25]. Moreover, pre-treatment with an anti-oxidant, *N*-acetylcysteine, reduced MDA levels and improved endothelial dysfunction after intravenous iron therapy in haemodialysis patients [26]. Cumulative burden of coronary risk factors leads to endothelial dysfunction, and FMD is one of the most widely used tools for the evaluation of endothelial function [27], [28]. These findings suggest that chelation of iron might decrease oxidative stress generation and subsequently improve endothelial dysfunction, making it a promising therapeutic strategy for preventing CVD in high-risk subjects such as smokers and renal failure patients.
